# Relation of Team Size and Success With Injuries and Illnesses During Eight International Outdoor Athletics Championships

**DOI:** 10.3389/fspor.2019.00008

**Published:** 2019-07-31

**Authors:** Pascal Edouard, Andy Richardson, Laurent Navarro, Vincent Gremeaux, Pedro Branco, Astrid Junge

**Affiliations:** ^1^Inter-University Laboratory of Human Movement Science (LIBM EA 7424), University of Lyon, University Jean Monnet, Saint Etienne, France; ^2^Sports Medicine Unit, Department of Clinical and Exercise Physiology, Faculty of Medicine, University Hospital of Saint-Etienne, Saint-Etienne, France; ^3^Medical Commission, French Athletics Federation (FFA), Paris, France; ^4^Division de Médecine Physique et Réadaptation, Swiss Olympic Medical Center, Centre de Médecine du Sport, Centre Hospitalier Universitaire Vaudois, Lausanne, Switzerland; ^5^European Athletics Medical & Anti Doping Commission, European Athletics Association (EAA), Lausanne, Switzerland; ^6^Institute of Cellular Medicine, Newcastle University, Newcastle Upon Tyne, United Kingdom; ^7^Mines Saint-Etienne, Univ Lyon, Univ Jean Monnet, INSERM, U 1059 Sainbiose, Centre CIS, Saint-Etienne, France; ^8^Institute of Sport Sciences, University of Lausanne, Lausanne, Switzerland; ^9^Health and Science Commission, International Association of Athletics Federations (IAAF), Monaco, Monaco; ^10^Medical School Hamburg, Hamburg, Germany; ^11^Swiss Concussion Center, Schulthess Clinic Zurich, Zurich, Switzerland

**Keywords:** health promotion, sports injury prevention, illness prevention, injury and illness surveillance, track and field, top-level athletes, injury risk

## Abstract

**Introduction:** The number of injuries and illnesses during major athletics championships vary according to sex and discipline. They may also differ between countries (national teams) given the differences in training, medical care, nutrition, lifestyle habits, and in travel to the championships. In addition, injuries and illnesses may influence the performance during the championships. Therefore, the aim was to analyse the differences in the injury and illness occurrence during international outdoor athletics championships with regards to the athlete's country, as well as establishing the potential relationships with the success of the country during the respective championships.

**Method:** The national medical teams and the local organizing committee physicians reported all injuries and illnesses daily on a standardized injury and illness report form during 4 World and 4 European outdoor championships from 2007 to 2018. Results were presented as number of registered athletes, injuries, illnesses and medals (absolute and per 1000 registered athletes), and for countries of different team size.

**Results:** During these 8 championships, a total of 219 different countries participated with a total of 13059 registered athletes who incurred 1315 injuries and 550 illnesses. The number of injuries and illnesses per championships varied between countries. Countries with higher numbers of registered athletes had a higher number of injuries and illnesses, as well as a higher number of medals and gold medals. There were significant positive correlations between number of injuries/illnesses and number of registered athletes, medals, gold medals. Injury and illness numbers per 1,000 registered athletes differed between countries and team sizes. Analyzing country participation grouped according to the number of registered athletes, there were significant negative correlations between injury/illness and medals/gold medals per 1,000 registered athletes.

**Conclusions:** Given the correlation between health problems and country size, we suggest that medical services provision and staff should be adapted to the team size. In groups of different country team sizes, lower number of injuries and illnesses per registered athletes were correlated with higher number of medals and gold medals per registered athletes, which can support that injury and illness prevention should be recognized as a win-win performance-prevention strategy.

## Introduction

Several variables can influence the athlete's performance (Morin et al., 2012; Zaras et al., 2016; Siart et al., 2017; Boccia et al., 2019; Gross et al., 2019; Loturco et al., 2019; Melin et al., 2019). Among them, being healthy, without any injury or illness, seems to be an important one (Hanstad et al., 2011; Hägglund et al., 2013; Raysmith and Drew, 2016; Drew et al., 2017). In male professional football, higher injury incidence and/or severity and lower match availability had a significant negative influence on performance in league play and in European cups (Hägglund et al., 2013). Raysmith and Drew (2016) reported that reduced participation in training due to injury and/or illness was associated with performance failure during major international athletics championships. In their systematic review, Drew et al. (2017) concluded that there was strong evidence that increased availability of team members/athletes decreased the risk of failure, and pre-competition and in-competition injuries both were associated with increased risk of failure. Finally, high illness rates were reported to be associated with the poor performance of the Norwegian team during the 2006 Winter Olympic Games (Hanstad et al., 2011). Since, currently only one study (Raysmith and Drew, 2016) analyzed relationships between injury/illness and performance in athletics, there is a need to improve this knowledge, especially in the context of major athletics championship. In addition, a potential relationship between health and performance in sport underscores the need for better understanding of injuries and illnesses, and for the development and implementation of prevention strategies (van Mechelen et al., 1992; Edouard et al., 2011, 2015a, 2018).

Several studies have analyzed the number and incidence of injuries and illnesses during major athletics championships (Alonso et al., 2009, 2010, 2012, 2015; Edouard et al., 2013, 2014a,b, 2015b,c, 2016a, 2019b; Feddermann-Demont et al., 2014; Timpka et al., 2017; Edouard et al., under revision). Feddermann-Demont et al. (2014) reported that on average 81 injuries per 1000 registered athletes occurred during 13 international championships. The number of injuries per registered athletes varied between different types of championships: higher for World than European championships, higher for outdoor than indoor championships, higher for adult than youth/junior championships) (Feddermann-Demont et al., 2014). In addition, Edouard et al. (2015b) reported that male athletes had 25% more injuries during 11 international athletics outdoor and indoor championships than female athletes. For both genders, the highest number of injuries per 1,000 registered athletes were observed in combined events (i.e., decathlon for male and heptathlon for female athletes during outdoor championships, and heptathlon for male and pentathlon for female athletes during indoors championships (Edouard et al., 2009, 2010), marathon and long distances (Edouard et al., under revision). Regarding illnesses, during 11 international championships including outdoors and indoors, 43 illnesses per 1,000 registered athletes were reported with significantly higher values for outdoor compared to indoor championships, and for endurance compared to explosive disciplines, without significant differences between male and female athletes (Edouard et al., 2019a,b).

These studies analyzed the occurrence of injuries and illnesses during major athletics championships with regard to the type of championships (outdoor vs. indoor), the athlete's sex (male vs. female athletes), and/or the athletics disciplines (endurance vs. explosive, or the nine athletics disciplines) (Alonso et al., 2009, 2010, 2012, 2015; Edouard et al., 2013, 2014a,b, 2015b,c, 2016a, 2019b; Feddermann-Demont et al., 2014; Timpka et al., 2017; Edouard et al., under revision). However, many other factors can also influence the occurrence of injuries and illnesses, given their multifactorial nature (van Mechelen et al., 1992; Meeuwisse et al., 2007; Bittencourt et al., 2016). One potential factor could be the home country of the athlete, since the training, physical conditioning, preparation of championships, medical care and availability, nutrition, lifestyle, culture, etc. may all vary between countries, and these differences may influence the risk of injuries and/or illnesses. In addition, international athletics championships often take place outside of the athlete's home country, and thus causing different travel requirements. Traveling may influence the risk of injuries and illnesses through travel itself, jet lag, changes in nutritional habits (foods and fluids), changes in environmental conditions (temperature, humidity, pollution, altitude, etc.), endemic pathogens, sanitation standards, and/or cultures different to the athlete's home country (Schwellnus et al., 2012; Fowler, 2016; Soligard et al., 2016; Mahadevan and Strehlow, 2017; Lohr et al., 2018; Schwellnus, 2019).

In this context, the aim of the present study was to analyse the differences between countries in the occurrence of injury and illness during international outdoor athletics championships, and to investigate the potential relationship with the success of the country during the respective championships.

## Methods

### Study Design and Procedure

We conducted a total population study analyzing the injury and illness data collected prospectively during eight international athletics outdoor championships:

– World Outdoor Championships (WOC) 2007 (Alonso et al., 2009), 2009 (Alonso et al., 2010), 2011 (Alonso et al., 2012), 2013 (Alonso et al., 2015);
– European Outdoor Championships (EOC) 2012 (Edouard et al., 2014b), 2014, 2016, 2018.

The study design, injury and illness definitions, methods and data collection procedures, were the same for all the 8 outdoor championships (Alonso et al., 2009, 2010, 2012; Edouard et al., 2013, 2014b, 2015b,c, 2016a, 2019b; Feddermann-Demont et al., 2014; Edouard et al., under revision).

### Data Collection

About 1 to 2 months before each championship, all teams/countries participating in the respective championship were informed about the study objective and modalities through emails from the organizing federation to the team leaders and the medical teams. The day before the start of each championship, the detail of the study procedure and data collection was presented to medical teams during a meeting. During the period of each championship, national medical teams (physicians and/or physiotherapists) and/or local organizing committee physicians (LOC) were asked to report daily all newly incurred injuries and illnesses on standardized injury and illness report forms available in paper or in electronic format, available in several languages (English, French, German, Spanish, Russian, Arabic, Chinese). Report forms were collected daily, and if report forms were missing the medical teams and LOC were contacted again. The issue of duplicate reporting was solved by the consensus of at least two members of the research group; information from the national team physician's report was preferred over the Local Organizing Committee (LOC) physician's report.

### Injury and Illness Definitions

Injuries were defined as “all musculoskeletal injuries (traumatic and overuse) and concussions newly incurred during competition or training regardless of the consequences with respect to the athlete's absence from competition or training” (Junge et al., 2008; Alonso et al., 2009; Feddermann-Demont et al., 2014; Timpka et al., 2014; Edouard et al., 2015b). Illness was defined as “any physical complaint unrelated to an injury and occurring during the championships” (Alonso et al., 2010, 2012; Edouard et al., 2013, 2014b, 2015c; Timpka et al., 2014). In cases where a single injury incident resulted in more than one injured body part and/or type of injury, each body part and/or type injury was counted as a separate injury (Edouard et al., 2015b, 2016a; Edouard et al., under revision).

### Confidentiality and Ethical Approval

All participants (i.e., athletes) were informed about the study aim and modalities via flyers distributed to each individual athlete at each championships and posters displayed in several locations, and if requested, could have their data removed from the subsequent analysis at any time without consequence. All records in the injury and illness database were anonymous, i.e., they cannot be associated with individual athletes.

The study was reviewed and approved by the Saint-Etienne University Hospital Ethical Committee (IORG0004981). Given the nature of the data collection (routine usual data collection, not systematic to all participants included) and the large sample size, the written informed consent for each individual participant was not required, by the Saint-Etienne University Hospital Ethical Committee, in accordance with the national legislation and the institutional requirements.

### Data Analyses

National medical team participation (number of national medical teams who returned at least one injury report form divided by the number of countries with a medical team, as not all teams may have a medical team present on site, in percentage), athletes' coverage (number of registered athletes from a country with a medical team present who participated in the injury surveillance study divided by the total number of registered athletes, in percentage), and response rate (number of report forms returned by the national medical teams participating in the injury surveillance study divided by the number of expected report forms (i.e., number of national medical teams participating multiplied by the number of championship days), in percentage) were reported according to Edouard et al. (2016b).

Descriptive data reported were the number of countries participating in at least one championships, the number of “country participation” (i.e., one country participating in one championship), and the respective numbers of registered athletes, injuries, illnesses, medals, and gold medals as well as these variables per 1,000 registered athletes (with 95% confidence intervals) for the total population (Feddermann-Demont et al., 2014; Timpka et al., 2014; Edouard et al., 2015b, 2016a, 2019b; Edouard et al., under revision).

The number of registered athletes was calculated by using the list of registered athletes provided by the International Association of Athletics Federations (IAAF) or the European Athletics Association (EAA) for each championship (i.e., if an athlete registered for more than one championship he/she counted for each championship) (Edouard et al., 2015b, 2016a, 2019b; Edouard et al., under revision). Country participations were grouped according to the number of registered athletes per country per championship: <10, 10–24, 25–49, 50–99, >100 (Junge et al., 2009; Soligard et al., 2017).

Number of medals, gold medals and country ranking were used as performance outcome (Raysmith et al., 2019). The number of medals and gold medals as well as the country ranking (i.e., ranking of the country at the championship according to the number of gold, silver and bronze medals) for each championship and country was collected using the IAAF (https://www.iaaf.org/home) and EAA (https://www.european-athletics.org) websites.

Pearson's correlation tests, or Spearman's correlation tests when distribution normality was not observed, were used to determine the correlation coefficient and their significance. Significance was accepted at *p* < 0.05. The correlation coefficients were interpreted using Hopkin's threshold: *r* = 1: perfect; 1 > *r* ≥ 0.90 nearly perfect; 0.90 > *r* ≥ 0.70 very large; 0.70 > *r* ≥ 0.50: large; 0.50 > *r* ≥ 0.30: moderate; 0.30 > *r* ≥ 0.10: small; 0.10 > *r*: trivial (Hopkins, 2002). Data were analyzed using Excel (Office, Microsoft^®^, 2017) and JASP (JASP Team software, Version 0.8.5.1, University of Amsterdam, Netherlands).

## Results

On average, 90.2% of all national medical teams, covering 81.9% of registered athletes, participated in the injury and illness surveillance project, and returned 90.5% of the expected report forms. No athlete refused to allow his/her data to be used for scientific research.

A total of 219 different countries participated in at least one of the eight international outdoor championships. All five continents were represented: 55 (25.1%) countries from Africa, 45 (20.5%) from North and South America, 46 (21.0%) from Asia, 53 (24.2%) from Europe, and 20 (9.1%) from Oceania. 20.5% (*n* = 45) of countries participated in all eight championships, three (1.4%) in seven, two (0.9%) in six, 134 (61.2%) in four, 21 (9.6%) in three, nine (4.1%) in two, and five (2.3%) one. This resulted in a total of 1015 country participations.

A total of 13059 athletes registered at the eight championships. The number of registered athletes per country and championship ranged from 1 to 138. For most of the country participations (*n* = 691; 68.1%), the number of registered athletes in a championship was below ten. It was between 10 and 24 athletes for 14.7% country participations, 25 to 49 (10.3%), 50 to 99 (5.6%), and ≥100 (1.3%) (Table 1).

**Table 1:** Number of countries participating in at least one championships, number of “country participation” (i.e., one country participating in one championship), numbers of registered athletes, injuries, illnesses, medals and gold medals, as well as injuries, illnesses, medals and gold medals per 1,000 registered athletes (with 95% confidence intervals) according to the number of registered athletes for each country participation (i.e., <10, 10–24, 25–49, 50–99, >100).

	**Number of athletes per country participation**					
	**<10**	**10–24**	**25–49**	**50–99**	**>100**	**All**
**NUMBER OF (n (%))**						
Countries	183 (83.6)	46 (21.0)	37 (16.9)	18 (8.2)	4 (1.8)	219^*^ (100.0)
Country participation	691 (68.1)	149 (14.7)	105 (10.3)	57 (5.6)	13 (1.3)	1015 (100.0)
Registered athletes	1712 (13.1)	2305 (17.7)	3748 (28.7)	3800 (29.1)	1494 (11.4)	13059 (100.0)
All injuries	218 (16.6)	252 (19.2)	408 (31.0)	340 (25.9)	97 (7.4)	1315 (100.0)
All illnesses	109 (19.8)	102 (18.5)	154 (28.0)	160 (29.1)	25 (4.5)	550 (100.0)
All medals	50 (5.4)	86 (9.3)	297 (32.2)	295 (32.0)	193 (21.0)	921 (100.0)
Gold medals	16 (5.8)	20 (7.2)	91 (32.9)	87 (31.4)	63 (22.7)	277 (100.0)
**NUMBER PER 1,000 REGISTERED ATHLETES (95%CI)**						
All injuries	127.3 (111.5 to 143.1)	109.3 (96.6 to 122.1)	108.9 (98.9 to 118.8)	89.5 (80.4 to 98.5)	64.9 (52.4 to 77.4)	100.7 (95.5 to 105.9)
All illnesses	63.7 (52.1 to 75.2)	44.3 (35.9 to 52.6)	41.1 (34.7 to 47.4)	42.1 (35.7 to 48.5)	16.7 (10.2 to 23.2)	42.1 (38.7 to 45.6)
All medals	29.2 (21.2 to 37.2)	37.3 (29.6 to 45.0)	79.2 (70.6 to 87.9)	77.6 (69.1 to 86.1)	129.2 (112.2 to 146.2)	70.5 (66.1 to 74.9)
Gold medals	9.3 (4.8 to 13.9)	8.7 (4.9 to 12.5)	24.3 (19.4 to 29.2)	22.9 (18.1 to 27.7)	42.2 (32.0 to 52.4)	21.2 (18.7 to 23.7)

* *the number of countries for “all” is different than the sum of the number of countries reported in each country participation group, since the number of athletes registered could have differed in the same country between championships*.

*There were significant negative correlations between the number of injuries per 1,000 registered athletes and the number of medals per 1,000 registered athletes (r = –0.92, p <0.001, nearly perfect) and of gold medals per 1,000 registered athletes (r = –0.90, p < 0.001, nearly perfect), and between the number of illnesses per 1,000 registered athletes and the number of medals per 1,000 registered athletes (r = –0.92, p < 0.001, nearly perfect) and of gold medals per 1,000 registered athletes (r = –0.99, p < 0.001, nearly perfect)*.

**Figure 1:**
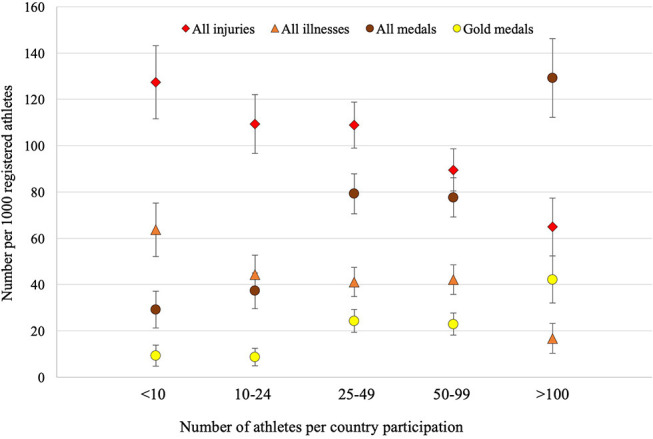
Number of injuries, illnesses, medals and gold medals per 1,000 registered athletes (and 95% confidence interval) per country participation according to the number of registered athletes for each country participation (i.e., <10, 10–24, 25–49, 50–99, >100). The number of injuries per 1,000 registered athletes correlated significantly negative with the number of medals (*r* = −0.92, *p* < 0.001, nearly perfect) and with the number of gold medals (*r* = −0.90, *p* < 0.001, nearly perfect) per 1,000 registered athletes. The number of illnesses per 1,000 registered athletes correlated significantly negative with the number of medals (*r* = −0.92, *p* < 0.001, nearly perfect) and of gold medals (*r* = −0.99, *p* < 0.001, nearly perfect) per 1,000 registered athletes.

A total of 1,315 injuries and 550 illnesses were reported during the eight championships. Between 0 and 23 injuries and between 0 and 18 illnesses were reported per country participation. The number of injuries per country participation varied from 0 to 2,000 injuries per 1,000 registered athletes, and the number of illnesses from 0 to 1,600 illnesses per 1,000 registered athletes, according to countries and championships. The number of injuries and illnesses per 1,000 registered athletes differed between country participation groups, with higher values in the group with small number of registered athletes and lower values in the group with high number of registered athletes (Table 1 and Figure 1).

A total of 921 medals and 277 gold medals were won during the eight championships. Seventy-six (34.7%) countries won at least one medal, and 52 (23.7%) one gold medal. The number of medals and gold medals per 1,000 registered athletes differed between country participation groups, with higher values in the groups with higher numbers of registered athletes, and lower values in the groups with smaller numbers of registered athletes (Table 1 and Figure 1).

Analyzing all country participations (*n* = 1015), significant positive correlations were observed between the number of injuries and the number of registered athletes (*r* = 0.666, *p* < 0.01, large), medals (*r* = 0.545, *p* < 0.01, large), gold medals (*r* = 0.475, *p* < 0.01, moderate), number of medals per 1,000 registered athletes (*r* = 0.233, *p* < 0.01, small), and a significant negative correlation between the number of injuries and the country ranking (*r* = −0.445, *p* < 0.01, moderate) (Table 2). There were significant positive correlations between the number of illnesses and the number of registered athletes (*r* = 0.493, *p* < 0.01, moderate), medals (*r* = 0.348, *p* < 0.01, moderate), gold medals (*r* = 0.306, *p* < 0.01, moderate), and a significant negative correlation between the number of injuries and the country ranking (*r* = −0.281, *p* < 0.01, small) (Table 2).

**Table 2:** Correlations between the numbers of injuries and illnesses with the numbers registered athletes, medals and gold medals, and country ranking, as well as correlations of these numbers reported per 1,000 registered athletes.

	**Number of registered athletes**	**Country ranking**	**Number of medals**	**Number of gold medals**	**Number of medals per 1,000 registered athletes**	**Number of gold medals per 1,000 registered athletes**
**NUMBER OF**						
All injuries	***r*** **=** **0.666**, ***p*** **<** **0.01, large**	***r*** **=** **−0.445**, ***p*** **<** **0.01, moderate**	***r*** **=** **0.545**, ***p*** **<** **0.01, large**	***r*** **=** **0.475**, ***p*** **<** **0.01, moderate**	***r*** **=** **0.233**, ***p*** **<** **0.01, small**	*r* = 0.145
All illnesses	***r*** **=** **0.493**, ***p*** **<** **0.01, moderate**	***r*** **=** **−0.281**, ***p*** **<** **0.01, small**	***r*** **=** **0.348**, ***p*** **<** **0.01, moderate**	***r*** **=** **0.306**, ***p*** **<** **0.01, moderate**	*r* = 0.118	*r* = 0.085
**NUMBER PER 1,000 REGISTERED ATHLETES**						
All injuries	*r* = −0.019	*r* = −0.003	*r* = 0.003	*r* = 0.002	*r* = 0.100	*r* = 0.025
All illnesses	*r* = −0.063	*r* = 0.117	*r* = −0.047	*R* = −0.034	*r* = −0.046	*r* = −0.001

*Significant correlations are highlighted in bold*.

Analyzing country participation groups (Table 1 and Figure 1), there were significant negative correlations between the number of injuries and the number of medals (*r* = −0.92, *p* < 0.001, nearly perfect) and of gold medals (*r* = −0.90, *p* < 0.001, nearly perfect) per 1,000 registered athletes, and between the number of illnesses and the number of medals (*r* = −0.92, *p* < 0.001, nearly perfect) and of gold medals (*r* = −0.99, *p* < 0.001, nearly perfect) per 1,000 registered athletes.

## Discussion

The main results of the present study were that (i) the number of injuries and illnesses per championship varied between countries, (ii) countries with higher number of registered athletes had a higher number of injuries and illnesses, as well as a higher number of medals and gold medals and a better country ranking, (iii) in country participations groups according to the number of registered athletes, lower number of injuries and illnesses per registered athletes were correlated with higher number of medals and gold medals per registered athletes.

### Injury and Illness Rates Varied Between Countries

During major athletics championships, the numbers and rates of injury and illness differed between countries. Many parameters may have contributed to these differences, such as “environmental” factors (training, nutrition, availability of medical services, travel to competition etc.) and the individual athlete's intrinsic characteristics. Moreover, these differences could be due to methodological aspects, e.g., differences in injury/illness surveillance participation, response rate, and willingness to provide injury/illness data between countries/medical teams.

By grouping our data according to the number of registered athletes per country participation, we found a significant correlation between the number of injury/illness per 1,000 registered and the number of registered athletes per country participation: countries with higher numbers of registered athletes in a championship reported lower injury/illness rates (Figure 1). This almost linear inverse relation between the size of the teams and injury rates has also been reported for the Summer Olympic Games 2008 (Junge et al., 2009), 2012 (Engebretsen et al., 2013), and 2016 (Soligard et al., 2017), and for the illness rates during the Summer Olympic Games 2012 (Engebretsen et al., 2013) and 2016 (Soligard et al., 2017). The fact that small teams come to the championships without any medical team, and may have difficulties accessing health professionals in the preparation for championships might be a possible explanation. However, such results require further research to better understand these differences between countries in injury/illness rates (e.g., analyzing, for small teams, the availability of medical services before the championships, and the behavior regarding the use of medical services during the championships), in order to design appropriate injury/illness prevention measures.

### “Nothing Ventured, Nothing Gained”

Our study revealed a significant positive correlation between the number of registered athletes per countries and the number of (gold) medals won, i.e., countries with a higher number of registered athletes were more likely to win medals during major international championships. In addition, a significant positive correlation between the number of registered athletes and the number of injuries and illnesses were observed, i.e., countries with a higher number of registered athletes were most likely to have injured or sick athletes in their teams. Thus, when increasing the number of athletes, it may increase the chance to have top-level athletes who can win medals, but also may increase the risk of incurring injuries and illnesses in the team. As a practical implication, we can suggest that larger teams should prepare for the championships with appropriate medical services and staff to encompass the higher risk of health problems.

### Lower Injury and Illness Rates Seem to Allow Higher Performance During Athletics Championships

Previous studies reported a relationship between health and sport performance (Hanstad et al., 2011; Hägglund et al., 2013; Raysmith and Drew, 2016; Drew et al., 2017). There was strong evidence that injury and/or illness can impact sporting success, especially increased availability of team members/athletes decreased the risk of failure, and pre-competition and in-competition injuries both were associated with increased risk of failure in reaching the performance goals (Drew et al., 2017). In a population of 33 international track and field athletes followed during 5 seasons, the loss in training availability due to injury and/or illness was associated with performance failure during major international championships (Raysmith and Drew, 2016). Our present study supports the relationships between injury and illness and performance during major international championships. It also provides some additional preliminary findings on the relationships of country size (i.e., number of registered athletes per championships) with health problem and performance. Consequently, we fully agree with practical recommendations of Drew et al. (2017):

– “Athlete health should be prioritized as a component of the integrated performance system;
– Multiple stakeholders (e.g., clinician, coach, sport scientist, the athlete) are accountable for both performance and the health;
– Sacrificing an athlete's safety resulting in injury or illness may also result in lower performance;
– Performance cannot be researched without consideration of the health status of the athlete both during competition and the period prior.”

Injury and illness prevention should be seen and included as a win-win performance-prevention strategy (Edouard et al., 2019a,b). These injury and illness prevention measures should be especially implemented in period prior to a major event, within the 6-month prior as suggested by Raysmith and Drew (2016), or at least during the month prior to the major event as suggested several studies (Alonso et al., 2015; Edouard et al., 2015c; Timpka et al., 2017), and during the event itself as suggested by our present results. This could also be one way to improve adherence, compliance and engagement in injury and illness prevention programmes (Drew et al., 2017).

### Methodological Considerations

To our best knowledge, this is the first study to analyse the relationships between injuries/illnesses and performance success during major athletics championships. Although some limitations detailed below should be acknowledged, these preliminary results allow hypothesizing that injury and illness prevention could be a way of improving athletics performance. These present findings extend those from previous epidemiological studies during international athletics championships (Alonso et al., 2009, 2010, 2012, 2015; Edouard et al., 2014b, 2015b, 2016a, 2019b; Feddermann-Demont et al., 2014; Timpka et al., 2017; Edouard et al., under revision) by showing a close relationship between health and performance of the athletes. These results are based on a good methodological quality, regarding team participation and response rates (Edouard et al., 2016b), and on a large sample size (219 countries, 13,059 registered athletes, 1,315 injuries and 550 illnesses) allowing more representative results.

In addition to the limitations previously discussed (Alonso et al., 2009, 2010, 2012; Edouard et al., 2013, 2014b, 2015b,c, 2016a, 2019b; Feddermann-Demont et al., 2014; Edouard et al., under revision), others should be acknowledged. The country participation (i.e., one country participating in one championship) means that some countries and athletes were counted more than once, e.g., in the groups of country participation according to the number of registered athletes (due to multiple participation of countries and athletes in several championships). The differences in numbers and rates of injuries/illnesses could be caused by different reporting of national medical teams. Especially, countries with a small number of registered athletes often do not have sufficient medical staffs and use the opportunity to consult the LOC medical centers for their injuries or illnesses. We reported the number of injuries and illnesses, which is different from the number of injury events, and of injured and sick athletes. Injury and illness information should not be interpreted as risk indicators at the level of individual athletes (Edouard et al., 2019b; Edouard et al., under revision). We chose number of medals and country ranking as sports performance outcome, which relates to the country and not to the individual athletes (e.g., metrics parameters, intra-personal parameters) (Drew et al., 2017; Raysmith et al., 2019). We acknowledge that numerous factors (e.g., physical, physiological, psychological, and/or technical aspects), in addition to injury and illness, may influence a performance outcome (Raysmith and Drew, 2016). The number of (gold) medals per country per championship collected on the IAAF and EAA websites for the present study could have changed from the time of the competition (and time of the injury/illness data collection), since some athletes who won medals in the championships have been excluded *a posteriori* because of doping.

## Conclusions

This study showed that the number of injuries and illnesses during eight major international athletic championships varied between countries with different numbers of registered athletes. Larger countries reported higher number of injuries and illness, and small countries reported higher injury and illness rates. Thus, medical services provision and staff need to be adapted to the size of the team, and small teams may need special support in their medical services provision and staff. Country participations with a higher number of registered athletes reported fewer injuries and illnesses per registered athlete, and won more medals and gold medals per registered athlete. This highlights that injury and illness prevention should be included as a win-win performance-prevention strategy.

## Data Availability

The raw data supporting the conclusions of this manuscript will be made available by the authors, after explicit and justified request, to any qualified researcher.

## Ethics Statement

The studies involving human participants were reviewed and approved by the study was reviewed and approved by the Saint-Etienne University Hospital Ethical Committee (IORG0004981). Written informed consent for participation was not required for this study in accordance with the national legislation and the institutional requirements.

## Author Contributions

PE: conceived and designed the present analysis. PE and PB: performed data collection. PE and LN: analyzed the data. PE, PB, and AJ: interpreted the results. PE: drafted the manuscript and prepared the table/figure. PE, AR, LN, VG, PB, and AJ: edited, critically revised the manuscript, and approved the final version.

### Conflict of Interest Statement

The authors declare that the research was conducted in the absence of any commercial or financial relationships that could be construed as a potential conflict of interest.
